# Measuring Biofouling Potential in SWRO Plants with a Flow-Cytometry-Based Bacterial Growth Potential Method

**DOI:** 10.3390/membranes11020076

**Published:** 2021-01-20

**Authors:** Nirajan Dhakal, Sergio G. Salinas-Rodriguez, Joshua Ampah, Jan C. Schippers, Maria D. Kennedy

**Affiliations:** 1 Water Supply Sanitation and Environmental Engineering Department, IHE Delft Institute for Water Education, Westvest 7, 2611 AX Delft, The Netherlands; s.salinas@un-ihe.org (S.G.S.-R.); joshbeckdon@yahoo.com (J.A.); jancschippers@gmail.com (J.C.S.); m.kennedy@un-ihe.org (M.D.K.); 2 Wetsus European Centre of Excellence for Sustainable Water Technology, Oostergoweg 9, 8911 MA Leeuwarden, The Netherlands; 3 Faculty of Civil Engineering, Delft University of Technology, Stevinweg 1, 2628 CN Delft, The Netherlands

**Keywords:** bacterial growth potential, flow cytometry, seawater reverse osmosis, pre-treatment, biofouling

## Abstract

Measuring the bacterial growth potential of seawater reverse osmosis (SWRO) feed water is an issue that is receiving growing attention. This study developed and demonstrated the applicability of the flow-cytometry (FCM)-based bacterial growth potential (BGP) method to assess the biofouling potential in SWRO systems using natural microbial consortium. This method is relatively fast (2–3 days) compared to conventional bioassays. The effect of the potential introduction of nutrients during measurement has been studied thoroughly to achieve the lowest measure value of about 45,000 cells/mL, which is equivalent to about (10 µg-C glucose/L). The BGP method was applied in two full-scale SWRO plants that included (i) dissolved air flotation (DAF) and ultra-filtration (UF); (ii) dual-media filtration (DMF) and cartridge filter (CF), which were compared with the cleaning frequency of the plants. A significant reduction (54%) in BGP was observed through DAF–UF as pre-treatment (with 0.5 mg Fe^3+^/L), while there was a 40% reduction by DMF–CF (with 0.8 mg Fe^3+^/L). In terms of the absolute number, the SWRO feed water after DAF–UF supports 1.5 × 10^6^ cells/mL, which is 1.25 times higher than after DMF–CF. This corresponds to the higher cleaning-in-place (CIP) frequency of SWRO with DAF–UF compared to DMF–CF as pre-treatment, indicating that the BGP method has an added value in monitoring the biofouling potential in SWRO systems.

## 1. Introduction

Biofouling in seawater reverse osmosis (SWRO) systems remains the major challenge for its cost-effective operation [1,2,3,4,5]. The main consequences of biofouling are (i) decreased membrane permeability, (ii) increased pressure drop along the spacer channel, resulting in increased frequency of chemical cleaning and possible increase in replacement frequency of membrane [4]. In practice, several methods for biofouling control have been investigated, such as the application of the pre-treatment prior to SWRO to remove bacteria and biodegradable organic matter [1], and dosing of biocides [6]. Detection of biofouling, while membranes remain in operation, is of great importance to avoid costly sacrifice of SWRO elements for autopsy [7]. 

A promising online detection of biofouling was attempted using a membrane fouling simulator (MFS) [8] and biofilm formation rate (BFR) [9]. Kurihara et al. [9] recently found a good correlation between the BFR and a chemical cleaning intervals in SWRO plants. However, the rate of biofouling measured as development of head loss across the feed/brine channel in MFS and BFR systems occurs at the same rate as in the full-scale SWRO plant. Thus, the detection of biofouling in an early stage using MFS and BFR is limited. Therefore, the application of bacterial growth potential (BGP) methods gained attention in the membrane-based desalination industry. The first assimilable organic carbon (AOC) method was developed by Van der Kooij et al. for freshwater and was measured by pasteurizing the sample (at 70 °C for 30 min), inoculating with *Pseudomonas fluorescens Pl 7* bacteria for a period of 14 days [10] and later by adding *Spirillum* sp. *NOX* together with *Pl 7* [11,12]. Lately, bacterial culture *(Flavobacteriumjohnsoniaestrain A3)* was introduced to utilize polysaccharides and proteins [13], but still the utilization of more complex compounds, such as polysaccharides and proteins, was a challenge. Therefore, to overcome the disadvantages of the above-mentioned approaches, an indigenous microbial consortium was used in AOC measurements, which broaden and diversify the substrate utilization range [14,15]. Ross et al. [15] showed that bacterial growth using an indigenous microbial consortium was 20% higher than when using pure culture strain.

In seawater, Weinrich et al. [16] and Jeong et al. [17] recently measured growth potential by using specific single bioluminescent strain bacteria *Vibrio fischeri* and *Vibrio harveyi*, respectively. These methods are very fast—1 h for the Weinrich method and 1–3 days for the Jeong method. The use of a single bacterial strain allows normalization of the yield based on a carbon source, enabling conversion of bacterial growth to a carbon concentration. However, a challenge of using the single strain is it may not reflect broader substrate utilization. These methods also cannot use natural bacteria as not all indigenous bacteria have the property of bioluminescence. Therefore, it is of great importance to develop a BGP method using natural consortium bacteria, which provide reliable information regarding biofouling potential in SWRO systems. 

Dixon et al. [7] and Quek et al. [18] used turbidity and a microbial electrolysis cell biosensor, respectively, to measure the bacterial growth potential in seawater using an indigenous microbial consortium. Recently, flow cytometry and the ATP-based BGP methods have been newly developed as fast, reliable, and accurate. Abushaban et al. [19,20,21,22] developed the ATP-based BGP method using natural consortium bacteria, which was applied in real SWRO desalination plants. The method is fast (2–3 days), sensitive with the limit of detection (LOD), and the ATP _direct method_ and ATP _filtration method_ are 0.3 ng-ATP/L, 0.06 ng-ATP/L, respectively. Table 1 summarizes the available growth potential methods that can be applied in seawater. In this study, we investigated the possibility of using the flow-cytometry-based bacterial growth potential method to assess the biofouling potential in SWRO systems. The flow-cytometry method (FCM) has been widely applied in freshwater [23] and its application, especially in seawater, has not been extensively studied. FCM can be used to measure total (live and dead) bacterial cells using nucleic-acid-targeting stains such as DAPI or SYBR^®^ Green I (St. Louis, MO, USA) [24,25].

FCM can distinguished the live and dead bacterial cells by staining with SYBR^®^ Green I and Propidium Iodide (PI). The dye PI penetrates bacterial cells with disrupted membranes while SYBR^®^ Green I can bind the nucleic acid of both live and dead bacterial cells [26]. Recently, Dixon et al. [7] and Farhat et al. [27] published a paper that describes the application of BGP using FCM and indigenous bacteria as inoculum for biofouling detection in SWRO membranes and different water types, respectively. The FCM-based growth potential assay using indigenous bacterial community demonstrated its potential for monitoring the biological stability of different waters compared to previously developed assays [7,27]. Moreover, the limit of detection of the methods and the effect of possible contamination that might originate from sample preparation, bottles, chemicals, pipette, etc., were not considered; this might overestimate the result and achieve higher limit of detection in the BGP method using FCM in seawater. Briefly, FCM has a maximum limitation of bacterial cell count (~10^7^ cells/mL) and, if exceeded, the sample needs to be diluted. Moreover, the effect of salinity on bacterial count using FCM needs to be studied, otherwise the result of bacterial shock while diluting the samples may underestimate the BGP results. 

The objective of this article is to further develop and demonstrate the applicability of using the FCM-based BGP method to assess the biofouling potential in the pre-treatment and feed of SWRO systems using natural consortium bacteria. The following aspects have been investigated and are described in this article:

- Verify the reproducibility and effect of salinity while enumerating marine bacterial cells using FCM;
- Verify the effect of the introduction of nutrients that might originate from chemicals and/or bottles during BGP method;
- Develop a calibration curve and the LOD of the measurement using both artificial and natural seawater using glucose as substrate;
- Measure bacterial growth potential along the pre-treatment train of an SWRO desalination plant using an indigenous bacterial consortium.

## 2. Materials and Methods

### 2.1. Glassware Preparation

All the glassware/vials/caps were first washed with lab detergent (Alconox Ultrasonic Cleaner, Alconox, St. Louis, MO, U.S.A.), rinsed three times with Milli-Q water, soaked overnight in 0.2 M HCl solution and again rinsed with Milli-Q water, and were air-dried. Finally, all the glassware/vials were heated in a muffle furnace at 550 °C for 6 h to remove all traces of organic material while the caps were bathed in 100 g/L sodium persulfate solution at 60 °C for 1 h and then rinsed with Milli-Q water and air-dried.

### 2.2. Preparation of Artificial Seawater (ASW)

ASW was prepared to produce the calibration line of the BGP method using glucose as substrate, based on the average concentration of the coastal North seawater. ASW was prepared by adding J.T. Baker analytical grade salts (Na_2_CO_3_, NaHCO_3_, CaCl_2_.2H_2_O, KCl, Na_2_SO_4_, MgCl_2_.6H_2_O, NaCl) in Milli-Q water (Table 2). The Milli-Q water (Millipore, Burlington, MA, U.S.A.) was produced from tap water purified via a series of treatment steps: reverse osmosis; electro deionization; granular-activated carbon adsorption; ultraviolet (UV) disinfection; 0.22 µm filtration.

### 2.3. Bacterial Growth Potential (BGP)

**Figure 1:**
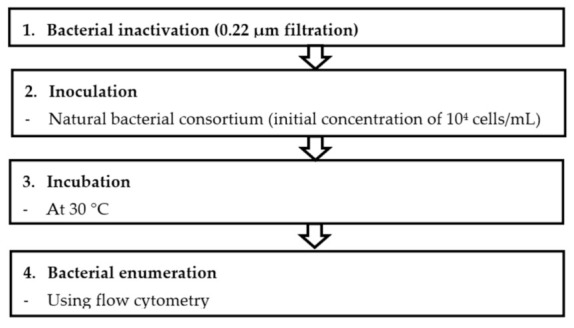
Procedure for measuring bacterial growth potential (BGP) in seawater using flow-cytometry method (FCM).

The BGP method comprises four major steps: bacterial inactivation, inoculation, incubation and bacterial enumeration as described in Figure 1.

The protocol involves filtration of a sample (V > 60 mL) through 0.22 µm PVDF filters to remove large particles and bacteria from seawater samples. Before the sample filtration, a 0.22 µm filter was flushed with Milli-Q water to remove the released carbon from the filter. The choice of the 0.22 µm filtration approach for the BGP test was based on the comparative study made with different approaches. The 0.22 µm filtered seawater sample (20 mL) was transferred into clean vials (in triplicate). A volume of 200 µL (equivalent to 10^4^ live cells/mL) of collected seawater from the North Sea (Jacobahaven, the Netherlands) was added to the vial containing 20 mL of sample. Samples were then incubated at a temperature of 30 °C [23] in order to achieve rapid bacterial growth and to reduce the time required to reach the maximum growth. A sample volume of 500 µL was taken from the incubated vials every 24 h, and the live bacterial cell concentration was enumerated using flow cytometry. The bacterial growth curves were then plotted, and the net live bacterial growth was calculated by subtracting the bacterial cell numbers at day zero (N_0_) from the maximum bacterial count (N_max_) during the incubation period. The net bacterial growth was considered as an indicator for BGP. 

#### 2.3.1. Reproducibility of FCM-Based BGP Method

North seawater collected from Jacobahaven, the Netherlands, was diluted (0–100%) using artificial seawater (Table 2). The ASW was autoclaved prior to dilution. The reproducibility of FCM was then determined by measuring the bacterial enumeration in diluted samples in triplicate. Likewise, all the BGP samples in this study were measured in triplicate to determine the reproducibility of the BGP method.

#### 2.3.2. Lowering the Limit of Detection of the BGP Method

The possible contamination that might originate from the bottles and chemicals used during the preparation of blank (artificial seawater) and its contribution to the BGP measurement was demonstrated considering the following three scenarios. For each scenario, the BGP was measured and compared. 

**Scenario 1:** ASW (35 g/L, pH 7.8) was prepared using salts NaCl, MgCl_2_.6H_2_O, Na_2_SO_4_, CaCl_2_.2H_2_O, KCl, NaHCO_3_, Na_2_CO_3_ and tested for the condition below to foresee the effect of bottle and chemical contamination on BGP.

- No heating of bottle and chemicals;
- Heating of bottle (550 °C, 6 h) and no heating of chemical;
- Heating of bottle and chemical (NaCl only) at 550 °C, 6 h.

**Scenario 2:** ASW (35 g/L, pH 5.5) was prepared using salts, NaCl only, to minimize the effect of chemical contamination from other salts. In this case, both the bottle and NaCl were heated at 550 °C, 6 h.

**Scenario 3:** ASW (35 g/L, pH 7.5) was prepared using salts, NaCl and NaHCO_3_. The chemical NaHCO_3_ was added to maintain the buffer capacity of the ASW. In this case, both the bottle and NaCl were heated at 550 °C, 6 h.

In all cases, heating of NaCl was only considered due to its higher melting point > 550 °C. 

#### 2.3.3. Effect of Salinity on Bacterial Enumeration by FCM 

To demonstrate the effect of salinity while enumerating seawater bacteria using FCM, the ASW (total dissolved solids—TDS = 35 g/L, Table 2) was prepared and diluted to different concentrations that ranged from 2 to 35 g/L using Milli-Q water. The bacterial enumeration in each sample was performed using flow cytometry.

#### 2.3.4. Calibration of the BGP Method

The BGP method was calibrated using glucose as substrate in both ASW and natural seawater samples. A glucose concentration that ranged from 0 to 2000 µg-C glucose/L was added in both water samples. All the samples were then spiked with a fixed concentration of 500 µg /L of *n* (NaNO_3_) and 100 µg/L of P (NaH_2_PO_4_). The BGP was measured using the protocol as described in Section 2.3. The calibration curves were plotted between the live net bacterial growth and the concentration range of glucose (0–2000 µg-C glucose/L). The yield factor of the tested natural bacterial consortium was calculated from the slope of the calibration curves. The equivalent carbon concentration was then calculated using the Equation (1) [28].

(1) $$\mathrm{ECC}\;\left[\left(\mu g\;C\right)L^{-1}\right]=\frac{\mathrm{Net}\;\mathrm{bacterial}\;\mathrm{growth}\;({\mathrm{cells}\;L}^{-1})}{\mathrm{Bacterial}\;\mathrm{specific}\;\mathrm{yield}\;({\mathrm{Cells}\;\mu g}^{-1})}$$

#### 2.3.5. Application of BGP Method in Full Scale SWRO Plants

The BGP method was applied to monitor the bacterial growth potential along the treatment process trains of full-scale desalination plants located in the Middle East. The raw seawater of both of the SWRO plants comes from open intakes and has similar characteristics to raw seawater properties. Some basic water quality parameters were salinity (69–71 mS/cm), TDS (~50 g/L), turbidity (4–10 NTU), water temperature (22–30 °C).

**Figure 2:**
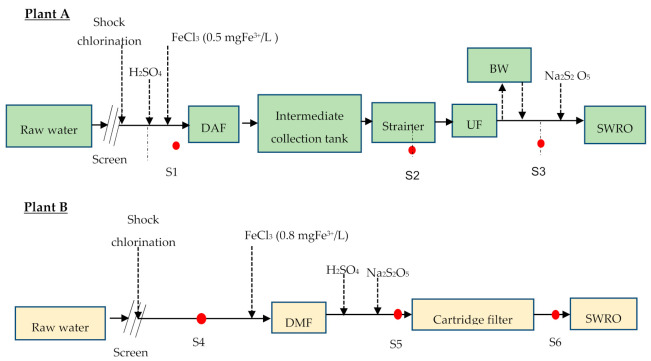
General scheme of (**A**) the dissolved air flotation/ultrafiltration/reverse osmosis (DAF–UF–RO) plant and (**B**) the dual media filtration/cartridge filter/reverse osmosis (DMF–CF–RO) plant (red dots are sampling points).

The general scheme of the plants included: (i) dissolved air flotation/ultrafiltration/reverse osmosis (DAF–UF–RO) and (ii) dual media filtration/cartridge filter/reverse osmosis (DMF–CF–RO) as shown in Figure 2a,b respectively. Both plants abstract the raw water through an open intake of about 7 m below the seawater surface, but from two different locations. In both intakes, shock chlorination (approximately 1 mg/L) was applied three times a day. The measured raw water pH was ~8.55, which was adjusted to approximately 7.90 (DAF–UF–RO) and 7.4 (DMF–CF–RO) by dosing H_2_SO_4_ in both plants. The coagulant (FeCl_3_) was continuously dosed in both plants at a concentration of 0.5 ppm of FeCl_3_ before DAF and 0.8 ppm of FeCl_3_ before DMF. The de-chlorination was performed before the SWRO unit by dosing Na_2_S_2_O_5_ (sodium metabisulfite). The dosing pump for Na_2_S_2_O_5_ was controlled based on the oxidation-reduction potential (ORP) value, which was set to a level of 250 mV. 

Brief specifications and operating conditions of the two plants are presented in Table 3.

As illustrated in Figure 2, samples (S1, S2, S3, S4, S5, and S6) were collected (one time) in clean glass bottles and transported to the Delft, the Netherlands, and measured using the BGP method. The samples were pasteurized at 70 °C, for 30 min on site before being transported to the Delft, the Netherlands. The historical (2015–2016) operational data for pressure drop (∆P) in RO before and after cleaning-in-place (CIP) for DAF–UF–RO plants were collected for two RO units (RO1 and RO2) and compared with the measured BGP of the RO feed water. The pressure drop (∆P) in RO of DMF–CF–RO plant was not collected as the plant was operated smoothly with no CIP for more than a year.

## 3. Results and Discussion

### 3.1. FCM for Enumerating Seawater Bacteria during BGP Method

**Figure 3:**
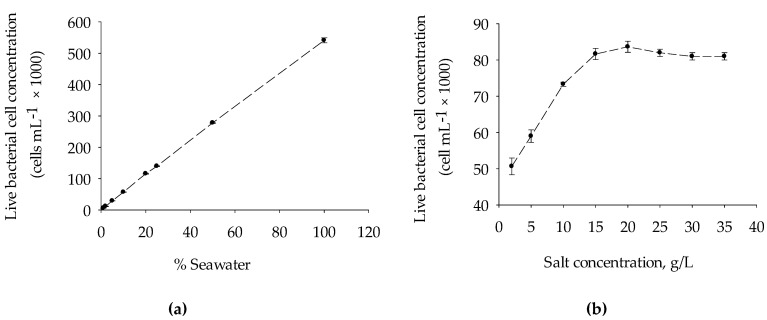
(**a**) Reproducibility and precision of flow cytometry for measuring live bacterial cell concentration in seawater sample and (**b**) bacterial live cell enumeration by FCM after inoculation of the same concentration seawater bacteria (collected from North seawater) to the ASW prepared at different concentrations (0–35 g/L) by varying the concentration of Na^+^ and Cl^−^ (Table 2).

Figure 3a shows the reproducibility of the FCM used in this study, as determined by the serial dilution of the North seawater using artificial seawater (35 g/L). A good linear relationship (R^2^ = 0.99) between the percentage of seawater and live bacterial cell concentrations was observed, with a percentage deviation that ranged from 0.6% to 9.1%. Furthermore, the effect of salinity when seawater bacteria were stained with fluorescence staining dye SYBR^®^ Green I (SG) and Propidium Iodide (PI) and enumerated using FCM was performed. As illustrated in Figure 3b, there was no substantial difference observed in the measured live bacterial cells concentration when the same concentration of seawater bacteria was inoculated in AWS (TDS ranged from 15 to 35 g/L). The live bacterial cell concentration declined at a rate of 2420 cell per g for TDS < 15 g/L, which could be attributed mainly to the effect of osmotic shock, which occurs when there is a sudden change in the solute concentration around bacterial cells. At a low level of salt concentration, water enters through the bacterial cells causing it to swell and finally burst [29]. The result elucidates the importance of diluting the seawater samples with same salinity artificial seawater (ASW) to avoid the effect of osmotic shock wherever necessary during FCM enumeration. Moreover, the organic carbon contamination that might originate from the bottle, chemicals, pipette, and laboratory environment during ASW (blank) preparation could influence the result of FCM and BGP methods. The effect of the introduction of nutrients that might originate from chemicals and/or bottles during the BGP method is discussed in Section 3.2.

### 3.2. Effect of Introduction of Nutrients Originated from Bottles and Chemicals on BGP

**Figure 4:**
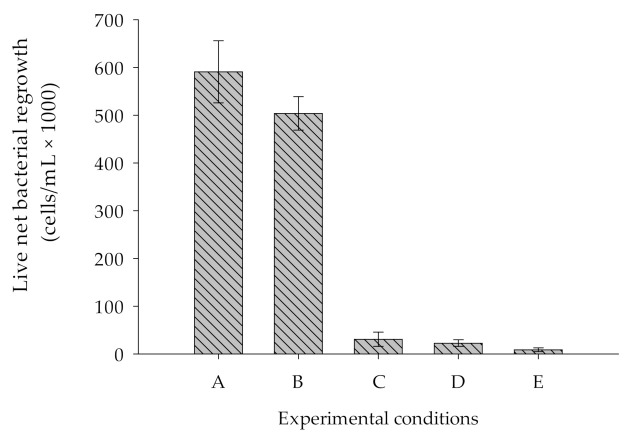
Comparison of live net bacterial regrowth in various ASW samples prepared with (**A**) all salts (pH = 7.8), in which both bottle and salts were not heated. (**B**) All salts (pH = 7.8), in which bottles were heated and salts were not heated. (**C**) All salts (pH = 7.8), in which both bottle and salt (NaCl only) were heated. (**D**) Two salts (NaCl + NaHCO_3_), in which both NaCl and bottle were heated. (**E**) One salt (NaCl), in which both NaCl and bottle were heated.

Figure 4 illustrates the comparative BGP measured in five different water samples. Samples A, B and C refer to all salts, i.e., NaCl, MgCl_2_.6H_2_O, Na_2_SO_4_, CaCl_2_.2H_2_O, KCl, NaHCO_3_, and Na_2_CO_3_.

The consortium of bacteria proliferated to approximately 600,000 ± 65,000 cells/mL in sample A (ASW prepared with all non-heated salts in a non-heated bottle). The higher growth in this sample was attributed to the introduction of nutrients originated from chemicals and bottles used during the preparation of ASW samples. To prove this, another test was performed with sample B (ASW prepared with all non-heated salts in a heated bottle at 550 °C for 6 h), which revealed that the live net bacterial growth was approximately 16% lower compared to that measured in sample A. Furthermore, a test performed with sample C (ASW prepared with all salts where NaCl and the bottle were heated at 550 °C for 6 h) showed substantial reduction (90%) in live net bacterial growth. The result illustrates that heating of the major salt (NaCl) and bottle in a muffle furnace at 550 °C for 6 h substantially reduced the effect of nutrients originated from bottle and chemicals added to prepare ASW on BGP measurements. As shown in Figure 4, sample E (ASW sample prepared with only NaCl (no other chemicals added), and both the chemical (NaCl) and bottle used were heated at 550 °C for 6 h) showed the lowest live net bacterial regrowth (8400 ± 4000 cells/mL), which was approximately 98.6% lower compared to sample A. Moreover, this could be due to the low pH (5.5) of the sample and to having no buffer capacity. Therefore, sample D (ASW sample prepared using NaCl and NaHCO_3_, where NaCl and bottle were heated at 550 °C for 6 h) showed the live net bacterial growth approximately to the level of 43,000 ± 12,000 cells/mL, which is approximately 92% lower than in sample A. While compared to the net bacterial growth in sample C and D, we observed no substantial difference despite the fact that in sample D no chemicals that constitute calcium and magnesium were added. Previous studies suggested that potassium, magnesium, and calcium are also essential elements required for the growth of marine bacteria [30,31]. As we did not observe the effect of adding calcium and magnesium in this study, we concluded that the blank prepared with ASW using two salts, namely, NaCl and NaHCO_3_, where chemical (NaCl) and bottle were heated at 550 °C for 6 h, substantially reduced the effect of nutrients that originate from chemicals and bottles during BGP measurement.

### 3.3. Calibration of BGP with Glucose as Substrate in ASW and Natural Seawater

**Figure 5:**
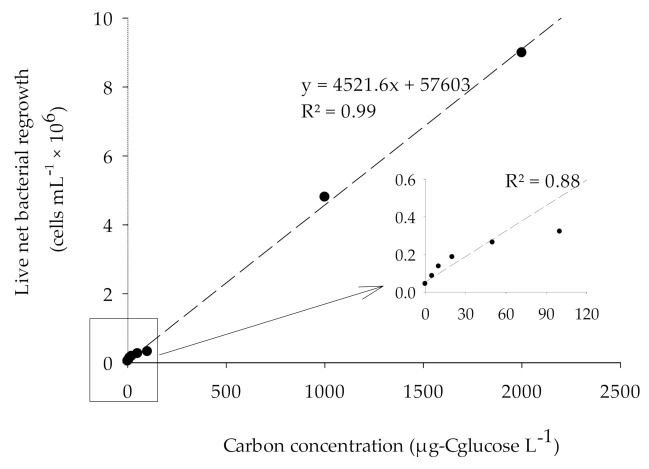
The correlation between the live bacterial net regrowth and carbon concentration (0–2000 µg-C_glucose_/L) in ASW sample.

**Figure 6:**
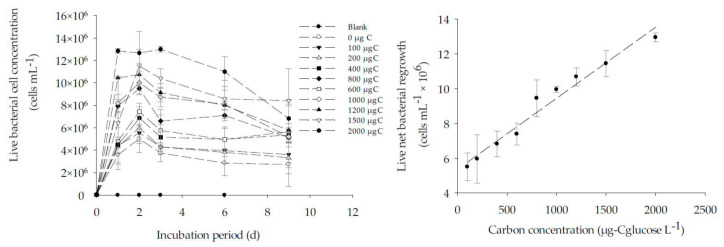
The correlation between the live bacterial net regrowth and carbon concentration (0–2000 µg-C_glucose_/L) in real seawater sample.

The result of BGP calibration performed with ASW and natural seawater and fortified with 0–2000 µg-C_glucose_/L is as shown in Figure 5 and Figure 6. The substrate glucose was chosen for the calibration of BGP as it has been reported that the glucose is a useful substrate during the characterization of a complex natural seawater microbial population [32]. As illustrated in Figure 5 and Figure 6, the response of an inoculated natural bacterial consortium to the utilization of glucose as a carbon source showed a linear fit with R^2^ > 0.95 for substrate concentration ranging from 0 to 2000 µg-C_glucose_/L in both ASW and natural seawater. While for lower range (0–100 µg-C_glucose_/L), R^2^ = 0.88 was observed (Figure 5). The specific yield of inoculated bacteria calculated from the slope of the calibration curve was approximately (4.4–4.6) × 10^6^ cells/µg-C. This was within the reported theoretical bacterial yield for *Pseudomonas fluorescens P−17* (4.1 × 10^6^ CFU /µg acetate-C) and *Spirillum* sp. *NOX* (1.2 × 10^7^ CFU/µg acetate-C) [33]. 

Using the calculated specific bacterial yield and the net bacterial growth, the equivalent carbon concentration (ECC) was calculated according to Equation (1) in both ASW and natural seawater (Table 4). Accordingly, the lowest measured value (LMV) for the BGP method was 43,000 ± 12,000 cells/mL, which is equivalent to a carbon concentration of 9.3 ± 2.6 µg-C_glucose_/L. The measured LMV for the BGP method was slightly lower than 10 µg-C acetate/L, a threshold value beyond which the biofouling is expected in freshwater [10]. The measured ECC in natural seawater when C = 0 was approximately 817 µg-C_glucose_/L. The higher measured ECC could be attributed to the occurrence of algal blooms in the seawater where the sample was collected. 

### 3.4. Measuring Biofouling Potential in Full Scale SWRO Plants Using the FCM-Based BGP Method

**Figure 7:**
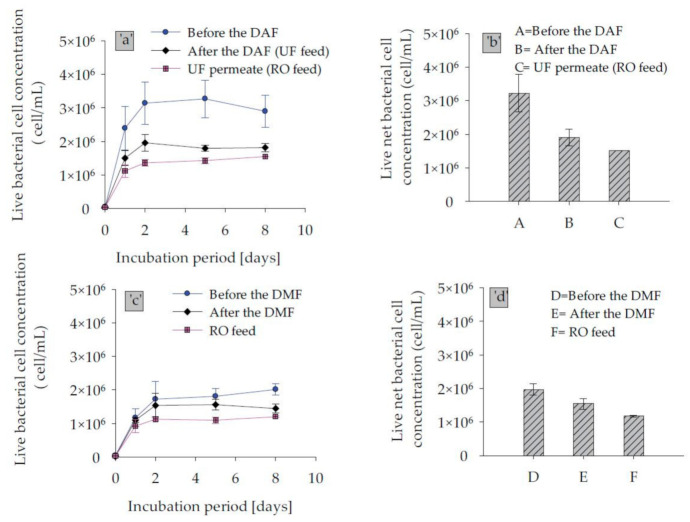
Live bacterial regrowth curve and calculated live net bacterial cell concentration from the growth curve for samples collected from (**a**,**b**) DAF–UF–RO, and (**c**,**d**) DMF–CF–RO treatment trains.

Biofouling potential was measured and compared over the treatment process trains of two desalination plants, which included DAF–UF–RO and DMF–CF–RO using the FCM-based BGP method. As illustrated in Figure 7, the measured net live bacterial growth potential of the raw water of the DAF–UF–RO treatment scheme, i.e., sample before DAF was approximately 1.6-fold higher than raw water of DMF–CF–RO line, i.e., sample before DMF. This variation could be due to the difference in raw water quality, as the abstractions of raw water was from two different intake locations. The result of liquid chromatography organic carbon detection (LC–OCD) of samples performed showed that raw water of DAF–UF–RO has slightly higher biopolymer fraction, i.e., 0.17 mg-C/L, compared to the raw water of DMF–CF–RO, i.e., 0.12 mg-C/L, while the fraction of low molecular weight acid was almost similar ( ~ 0.12 mg-C/L) in raw water of both plants. Therefore, the difference in biopolymer fraction, although not substantial, could be one of the reasons for higher net bacterial growth. Passow (2002) also reported that the biopolymer fraction could be degraded by bacteria in a matter of a few hours to several months [34]. 

Furthermore, the pre-treatment option DAF–UF showed a reduction of 54% in net bacterial growth, while it was only 40% by DMF–CF. This could be due to the higher removal of the biodegradable organic matter in dissolved air flotation operated with 0.5 mg/L of FeCl_3_ coagulant and followed by ultrafiltration. Moreover, in terms of an absolute number of bacterial growth, the SWRO feed water after DAF and UF supports 1.5 × 10^6^ cells/mL, which is 1.25 times higher than in SWRO feed water of DMF–CF, as illustrated in Figure 7b,d. This suggested that SWRO after DAF–UF is more vulnerable to biofouling compared to SWRO after DMF–CF. However, it should be noted that the raw water for DMF–CF had a lower net bacterial cell number to start with. 

**Figure 8:**
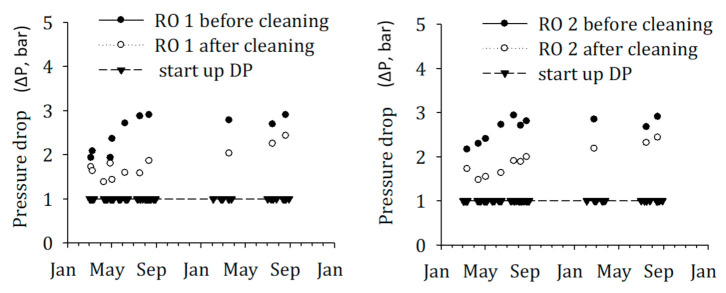
Historical (2015–2016), operational data for pressure drop (∆P) in RO before and after CIP.

Nevertheless, other water quality parameters measured for SWRO feed water of the DAF–UF–RO and DMF–CF–RO schemes during the time of the study showed very low fouling potential, as shown in Table 5. However, a remarkable increase in pressure drop was observed on the SWRO of the DAF–UF–RO system, which demands higher frequency of cleaning-in-place (CIP). In contrast, the SWRO in the scheme, DMF–CF–RO, was operated with no CIP for more than a year. Therefore, the historical SWRO operational data of DAF–UF–RO were collected and analyzed to answer the mystery of the higher increase in pressure drop (Figure 8). 

As illustrated in Figure 8, the increase in pressure drop (∆P) in SWRO units shows a steady growth after each cleaning-in-place (CIP) performed, and currently is at a level that only a marginal increase in ∆P requires CIP again. It is a clear indication of biofouling in RO membranes because particulate fouling can be excluded due to the pre-treatment of the feed water with ultrafiltration as demonstrated by the measured SDI and MFI–UF values of RO feed water (Table 5). The remaining high head loss after CIP is the main reason for the relatively rapid increase in head loss, arriving rapidly at a level that requires CIP to be repeated. The fundamental reason for the occurrence of biofouling in the membranes is the presence of biodegradable organic matter [39]. The biodegradable organic matter could have been introduced into the treatment process by the impure chemicals dosed, namely, sulfuric acid, sodium metabisulfite, ferric (coagulant). These compounds might have been biodegraded in the DMF of the DMF–CF–RO scheme, but not in the DAF–UF–RO scheme. The DAF–UF–RO scheme has no treatment step, which incorporates biodegradation on non-polymeric compounds. There is evidence that biologically active sand filtration RO significantly enhanced the RO membrane performance [40], presumably by removing the biodegradable organic matter [39]. In addition, it is possible that biodegradable organic compounds soon after the start-up of the DAF–UF–RO plant were present in the raw water and were not adequately removed in the DAF–UF–RO scheme due to the absence of a treatment process incorporating biodegradation. These biodegradable compounds probably originated from algal bloom(s). The previous study also demonstrated higher bacterial growth potential in a sample that has a higher concentration of algal organic matter [41].

## 4. Conclusions

- An FCM-based BGP method for seawater using natural microbial consortium as inoculum was developed and applied in full-scale SWRO plants. The developed method was relatively fast (2–3 days) to monitor the biofouling potential of pre-treatment and SWRO feed water.
- The percentage deviation on the reproducibility of the FCM measurement was below 10% and the variation in the FCM-based BGP method was approximately <5% and <20% when the method was applied for ASW and natural seawater, respectively.
- The effect of nutrients on the BGP method that originated from the bottle and chemicals was substantially reduced by 92% when blank (ASW) was prepared using NaCl and NaHCO_3_, where bottles and NaCl were heated at 550 °C for 6 h. With this approach, the lowest measured value of the FCM-based BGP method was approximately 10 µg-C_glucose_/L.
- The FCM-based BGP method showed good linear correlation (R^2^ ~ 0.9) between carbon concentration (0–2000 µg-C_glucose_/L) and live net bacterial growth, in both artificial and natural seawater.
- The method was applied to measure the bacterial growth potential through pre-treatment trains of two SWRO desalination plants in the Middle East. A significant reduction (54%) in bacterial growth potential was noticed through DAF–UF as pre-treatment (with 0.5 mg Fe^3+^/L), while it was 40% with DMF–CF (with 0.8 mg Fe^3+^/L).
- The absolute number of bacterial growth supported by the SWRO feed water after DAF–UF was approximately 1.25 times higher than SWRO feed water after DMF–CF. This corresponds to the higher CIP frequency of SWRO with DAF–UF as pre-treatment, suggesting that the FCM-based BGP method is a promising tool for measuring the biofouling potential in SWRO feed water. However, more experiments are required to develop a sound relationship between the BGP and the pressure drop increase in SWRO plants.

## Figures and Tables

**Table 1:** Bacterial growth potential methods for seawater application.

Author/Reference	Bacterial Inactivation	Bacterial Inoculation	Bacteria Enumeration Method	Detection Limit
Weinrich et al., (2011) [16]	Pasteurization (70 °C), 30 min	*V. harveyi*	Luminescence	<10 µg/L acetate
Dixon et al., (2012) [7]	Filtration (0.2 µm)	natural consortium bacteria	Turbidity	
Jeong et al., (2013) [17]	Pasteurization (70 °C), 30 min	*V. fischeri*	Luminescence	0.1 µg-C-glucose/L
Quek et al., (2015) [18]	-	natural consortium bacteria	Microbial electrolysis cell biosensor	
Abushaban et al., (2017) [19]	Pasteurization (70 °C), 30 min	natural consortium bacteria	Microbial ATP (direct method)	0.3 ng-ATP/L
Abushaban et al., (2019) [20]	Pasteurization (70 °C), 30 min	natural consortium bacteria	Microbial ATP (filtration method)	0.06 ng-ATP/L

**Table 2:** Inorganic ion composition of model artificial seawater (ASW).

Inorganic Ions	Concentration (g/L)
Chlorine (Cl^−^)	18.85
Sodium (Na^+^)	10.75
Sulphate (SO_4_^2−^)	2.69
Magnesium (Mg^2+^)	1.17
Calcium (Ca^2+)^	0.30
Potassium (K^+^)	0.38
Hydrogen Carbonate (HCO_3_^−^)	0.15
Total dissolved solids (TDS)	34.29

**Table 3:** Operating conditions of the three in seawater reverse osmosis (SWRO) desalination plants.

	*Plant A*	*Plant B*
Pre-treatment	- Coagulation + dissolved air flotation + ultrafiltration + cartridge filtration	- Coagulation + dual media filtration + cartridge filtration
Feed water pH adjustment	- From 8.55 to 7.9 by dosing H_2_SO_4_ in intake	- From 8.55 to 7.4 by dosing H_2_SO_4_ in RO feed
Coagulant dose (mgFe^3+^/L)	- 0.5	- 0.8
Ultrafiltration	- Vertical type	-
Media filtration	-	- Dual media (anthracite and sand); depth 1 m; filtration cycle = 24–48 h; contact time = 4–5 min
Filtration rate (m/h)	- 0.06 (UF flux = 60 L/m^2^.h)	- 11–14
SWRO recovery	- 40%	- 40%

**Table 4:** Maximum bacterial cell concentration (N_max_) and equivalent carbon concentration (ECC) in artificial and natural seawater.

Carbon Concentration, µg-C_glucose_L^−1^	Artificial Seawater		Natural Seawater	
	Maximum Live Net Bacterial Cell, N_max_ (×10^4^) (cells/mL) ^a^	Equivalent Carbon Concentration (µg-C_glucose_L^−1^)	Maximum Live Net Bacterial Cell, N_max_ (×10^5^) (cells/mL) ^a^	Equivalent * Carbon Concentration (µg-C_glucose_L^−1^)
0	4.3 ± 0.1	9.3	37.6 ± 8	817
5	8.6 ± 0.07	18.7	*n*.m.	*n*.m.
10	13.8 ± 1.13	30.0	*n*.m.	*n*.m.
20	18.7 ± 1.84	40.6	*n*.m.	*n*.m.
50	26.5 ± 1.06	57.6	*n*.m.	*n*.m.
100	32.2 ± 4.60	70.0	55.5 ± 8	443
200	*n*.m.	*n*.m.	60 ± 14	546
400	*n*.m.	*n*.m.	68.0 ± 7.4	728
600	*n*.m.	*n*.m.	74.3 ± 6.2	871
800	*n*.m.	*n*.m.	95 ± 10.6	1341
1000	480.6 ± 1.84	1045	100 ± 1.8	1455
1200	*n*.m.		107 ± 5.2	1614
1500	*n*.m.		115 ± 7.5	1796
2000	899.6 ± 7.00	1956	130 ± 2.5	2137

^a^ Values are average ± standard deviation; *n* = 3, *n*.m. = not measured. * The calculated ECC for natural seawater is blank corrected.

**Table 5:** RO feed water quality measured regarding SDI, MFI–UF, LC–OCD, and TEP.

Parameters	Units	RO Feed Water of	
		DAF–UF–RO	DMF–CF–RO
Silt Density Index (SDI) ^1^		<1.7	~1.5
Membrane fouling potential (MFI–UF_10kDa_) ^2^	s/L^2^	690	Bdl
Biopolymer concentration ^3^	mg-C/L	0.09	0.1
Transparent exopolymer particles (TEP_10kDa_) ^4^	mgXeq/L	0.06	0.04

^1^ SDI method [35]. ^2^ MFI–UF method [36]. ^3^ Biopolymer concentration [37]. ^4^ Transparent exopolymer particles (TEP) method [38].
